# Studies of Protein Wastes Adsorption by Chitosan-Modified Nanofibers Decorated with Dye Wastes in Batch and Continuous Flow Processes: Potential Environmental Applications

**DOI:** 10.3390/membranes12080759

**Published:** 2022-08-01

**Authors:** Dai-Lun Cai, Dinh Thi Hong Thanh, Pau-Loke Show, Su-Chun How, Chen-Yaw Chiu, Michael Hsu, Shir Reen Chia, Kuei-Hsiang Chen, Yu-Kaung Chang

**Affiliations:** 1 Department of Chemical Engineering, Ming Chi University of Technology, New Taipei City 243303, Taiwan; m09138210@mail2.mcut.edu.tw (D.-L.C.); chenyaw.chiu@gmail.com (C.-Y.C.); 2 Department of Safety, Health and Environmental Engineering, Ming Chi University of Technology, New Taipei City 243303, Taiwan; thanhdth213@gmail.com; 3 Zhejiang Provincial Key Laboratory for Subtropical Water Environment and Marine Biological Resources Protection, Wenzhou University, Wenzhou 325035, China; showpauloke@gmail.com; 4 Department of Chemical and Environmental Engineering, Faculty of Science and Engineering, University of Nottingham Malaysia, Semenyih 43500, Malaysia; 5 Department of Chemical Engineering and Biotechnology, Tatung University, Taipei 10452, Taiwan; schow@gm.ttu.edu.tw; 6 Chemist Scientific Corp., Taishan Dist., New Taipei City 243303, Taiwan; chemist7153@gmail.com; 7 Institute of Sustainable Energy, Universiti Tenaga Nasional, Jalan IKRAM-UNITEN, Kajang 43000, Malaysia; shireen.chia127@gmail.com

**Keywords:** lysozyme, removal, reactive green 19 dye, chitosan, nanofiber membrane

## Abstract

In this study, reactive green 19 dye from wastewater was immobilized on the functionalized chitosan nanofiber membranes to treat soluble microbial proteins in biological wastewater. Polyacrylonitrile nanofiber membrane (PAN) was prepared by the electrospinning technique. After heat treatment, alkaline hydrolysis, and chemically grafted with chitosan to obtain modified chitosan nanofibers (P-COOH-CS), and finally immobilized with RG19 dye, dyed nanofibers were generated (P-COOH-CS-RG19). The synthesis of P-COOH-CS and P-COOH-CS-RG19 are novel materials for protein adsorption that are not deeply investigated currently, with each of the material functions based on their properties in significantly improving the adsorption efficiency. The nanofiber membrane shows good adsorption capacity and great recycling performance, while the application of chitosan and dye acts as the crosslinker in the nanofiber membrane and consists of various functional groups to enhance the adsorption of protein. The dyed nanofibers were applied for the batch adsorption of soluble protein (i.e., lysozyme), and the process parameters including chitosan’s molecular weight, coupling pH, chitosan concentration, dye pH, dye concentration, and lysozyme pH were studied. The results showed that the molecular weight of chitosan was 50 kDa, pH 5, concentration 0.5%, initial concentration of dye at 1 mg/mL dye and pH 12, lysozyme solution at 2 mg/mL at pH 8, and the maximum adsorption capacity was 1293.66 mg/g at a temperature of 318 K. Furthermore, thermodynamic, and kinetic studies suggested that the adsorption behavior of lysozyme followed the Langmuir adsorption isotherm model and the pseudo-second-order kinetic model. The optimal adsorption and desorption conditions based on batch experiments were directly applied to remove lysozyme in a continuous operation. This study demonstrated the potential of dyed nanofibers as an efficient adsorbent to remove approximately 100% of lysozyme from the simulated biological wastewater.

## 1. Introduction

Various dye wastewaters are discharged from the textile industry. Among them, reactive and basic dyes are the main ones. It was reported that approximately 10–50% of reactive dyes are lost from the dyeing process into the effluent [1]. The dye wastewater usually contains hydrolyzed reactive dyes (30–60 g/L), dyeing auxiliaries, and electrolytes (60–100 g/L of NaCl and Na_2_CO_3_). The high salinity of dye wastewater has resulted in a high pH value of wastewater, i.e., at pH 10–11. Wastewater-containing dyes are very difficult to treat because they are relatively stable to light, heat, and oxidizing agents [2,3]. Therefore, it is necessary to remove or recover these reactive dyes from wastewater.

On the other hand, biological wastewaters were found to have detrimental impacts on the environment when they are discharged to receiving water bodies without proper treatment. The biological wastewaters contain extracellular polymers and soluble microbial products which leads to unpleasant odor and unfavorable color transformation [4,5,6]. Besides, an effective wastewater treatment requires large amount of disinfectant and coagulant and subsequently leads to increment of pollutant content and microbial growth in the processed wastewater [7]. Hence, there is an urgent demand of efficient techniques to remove water-soluble proteins from biological wastewater.

In our previous studies [6,8], the utilisation of eggshell waste in removing heavy metals, soluble chicken egg white (CEW) proteins, and dyes sequentially were concluded as competent pre-treatment for membrane filtration and bioremediation approach. The complex particles incorporating CEW and eggshell could remove dyes and use repeatedly, due to their good regeneration ability. In addition, it has showed good pseudo-chelating adsorption capability in eliminating soluble proteins before membrane filtration. It was concluded that eggshell-metal complexes exhibit high stability as adsorbent and possess low probability of secondary pollution. 

On the other hand, the ion-exchange adsorption for proteins is preferrable to use porous extrudate-shaped zeolite as adsorbent as it is selective for such application [9,10]. Recently, the investigation of adsorption behaviour of soluble proteins using NaY zeolite with immobilized metal ions was performed [11]. A pseudo metal ion-chelating adsorbent was produced through the encapsulation of the heavy metal ion waste on NaY zeolite to remove BSA. The Cu/NaY zeolite complex was concluded as a promising approach to treat water-soluble proteins economically.

Reactive dyes as pseudo-affinity ligands have widespread applications in protein purifications [12]. They are cheap, relatively safe, and can be used in large-scale purification processes. Some reactive dyes (e.g., Reactive Red 141, Reactive Orange 4, and Reactive Green 19 dyes) immobilized various nanofibers have been applied in the purification of various proteins [13,14,15,16]. They can be successfully applied to protein purification mainly because the dye molecules have various functional groups (e.g., hydrophilic, hydrophobic, and −SO_3_^−^) that have a specific force on protein molecules. The application of chitosan (CS) and reactive dyes (e.g., RG19 dye with six −SO_3_^−^ groups) in nanofiber membrane was previously studied as a crosslinker [17]. The CS-modified nanofiber membrane can be coupled with reactive dyes directly where the occurrence of reactive chlorine’s displacement was observed through bimolecular nucleophilic substitution under alkaline conditions [12]. As demonstrated in our previous study [17], the presence of negatively-charged reactive dyes on the nanofiber effects the uptake capacity of positively-charged PHMB due to the interaction between the PHMB and the −SO_3_^−^ groups in the dye molecules. In this work, wastewater containing RG19 dye and high salts (i.e., 20% NaCl and 10% Na_2_CO_3_) at pH 12 was used as a synthetic dye waste model. 

Based on the functional groups on the dye molecules, they may be applicable to removing the soluble microbial products (e.g., metabolic products, carbohydrate polymers, and water-soluble proteins) from the biological wastewater [1,18]. Hence, in this work, we attempted to use RG19 dye immobilized onto the CS-modified nanofiber to remove lysozyme as a model protein. The adsorption capacity of P-COOH-CS-RG19 was investigated with respect to protein removal from aqueous solutions. Lysozyme was used as a model protein. Lysozyme is an extensively studied protein and its physicochemical characteristics (e.g., structure, charge distribution, hydrophilicity/hydrophobicity) have been well known [18]. To account for various environmental factors related to water treatment, the optimization for lysozyme treatment has been conducted with a series of experiments, including coupling CS molecular weight, CS concentration and pH, RG19 immobilization pH and concentration, adsorption pH for lysozyme, and elution of adsorbed lysozyme. Simple Kinetic models (pseudo-first-order, pseudo-second-order, Elovich, and intraparticle diffusion models) [19,20,21] and equilibrium isotherm models (e.g., Langmuir, Freundlich, and Temkin models) [19,22] was used to describe the experimental results, in order to provide a better understanding on the kinetic rate and equilibrium parameters for lysozyme adsorption to the P-COOH-CS-RG19 nanofiber membranes. Moreover, continuous membrane chromatography was used to remove lysozyme under different conditions (e.g., pH, lysozyme concentration, and liquid flow rate). 

## 2. Experimental

### 2.1. Materials 

PAN yarn with a molecular weight of 1.2 × 10^5^ g/mol was acquired from Fortune Industries Inc., (Taichung, Taiwan). Different molecular weights of chitosan (CS) (50 KDa, water-soluble; 210 kDa and 340 kDa, soluble in 1% CH_3_COOH) were obtained from Charming & Beauty Co., Ltd., (Taipei, Taiwan), while Freudenberg Far Eastern Spunweb Co. Ltd., (Taoyuan, Taiwan) provided polyethylene terephthalate (PET) spun-bond fabric. Chemicals such as 1-ethyl-3-(3-dimethyl aminopropyl) carbodiimide (EDC) and 2-(N-morpholino) ethane sulfonic acid hydrate (MES) were obtained from Sigma-Aldrich, MO while lysozyme, acid orange 7 (AO7) (C_16_H_11_N_2_NaO_4_S) and toluidine blue O (TBO) (C_15_H_16_N_3_SCl) were provided from Sigma-Aldrich, (St. Louis, MO, USA). Sodium hydroxide (NaOH) and sodium acetate (CH_3_COONa) were supplied by Showa. Reactive Green 19 was obtained from First Chemical Co., Ltd, (Taipei, Taiwan). The Bradford reagent was obtained from Pierce Chemical Co., (Rockford, IL, USA). Sodium phosphate dibasic (Na_2_HPO_4_), sodium phosphate monobasic (NaH_2_PO_4_) and glycine were provided from J.T. Baker (Radnor, NJ, USA).

### 2.2. Preparation of Dyed Nanofiber Membrane

**Figure 1:**
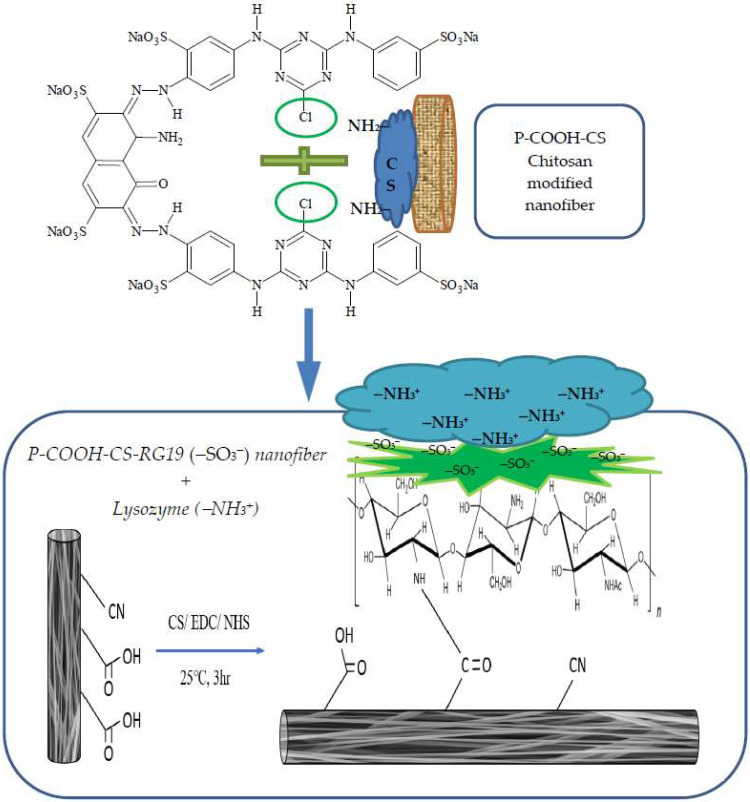
Illustrates the attachment of lysozyme onto the P-COOH-CS-RG19 nanofiber membrane.

An electrospinning device was used to synthesize the nanofiber membranes (Falco Tech Enterprise Co., Ltd., New Taipei City, Taiwan) [16]. Figure 1 displays the structure of the P-COOH-CS-RG19. Previous studied have reported the detailed methodology of P-COOH and P-COOH-CS synthesis [15,23]. The alkaline hydrolysis-treated PAN nanofiber membrane is labelled as P-COOH, while the coupling of CS with P-COOH is P-COOH-CS. First, the P-COOH-CS was immersed in a 5 mL solution consists of 3 mg/mL RG19 dye in 20% NaCl and 10% Na_2_CO_3_ solution, and subsequently stirred for 3 h at 70 °C and 150 rpm. The nanofiber membrane was incubated in 5 mL distilled water for 30 min at 70 °C to wash away the unreacted dye residues and this step was repeated for three times. Determination on the dye molecules immobilized on the nanofiber membrane was performed by UV-Vis spectrophotometer to measure the maximum absorption wavelength of RG19 dye at 630 nm.

### 2.3. Characterization of Nanofiber Membranes

Several analyses were performed to characterize nanofiber membranes, which are SEM, FTIR and TGA analysis. SEM analysis (Hitachi S-2600 H, Tokyo, Japan) was used to investigate the diameter and morphology of nanofiber mats, for samples coated with platinum prior analysis. The FTIR analysis (Perkin Elmer Spectrum One, Shelton, CT, USA) was performed over the wavenumber ranging from 400–4000 cm^−1^. TGA analysis (Mettler Toledo, Q600 New Castle, DE, USA) was conducted at a heating rate of 20 °C/min using thermogravimetric analyzer with the condition under nitrogen purging. An UV-Vis spectrophotometer (GE Healthcare Biosciences Ultraspec 3300 Pro, Buckinghamshire, UK) was used to investigate the concentrations of AO7 and TBO dyes at 485 nm and 633 nm, respectively [16,24]. The determination for the carboxylic and amine groups’ content on the P-COOH and P-COOH-CS nanofiber membranes were performed as well. 

### 2.4. Lysozyme Removal Study

The batch adsorption study was performed using P-COOH-CS-RG19 in a single-factored experimental setup. About 0.3 g of membrane sample was contacted with a fixed concentration of lysozyme solution at 100 rpm constantly. The single-factored experiments were conducted by changing the chitosan coupling pH (pH 4–8) and chitosan coupling concentration (0.1–5.0%), RG19 dye immobilization pH (pH 8–12), RG19 dye initial concentration (0.5–5.0) and lysozyme adsorption pH (pH 4–12), lysozyme adsorption concentration (0.5–2.0 mg/mL), and lysozyme solution temperature (288–318 K). The lysozyme concentration in the liquid phase was measured at 280 nm using UV-Vis spectrophotometer and the extinction coefficient of 2.65 mL/mg·cm [22].

For kinetic studies, 125 mL flasks containing a mixture of nanofiber (0.03 g) and lysozyme (50 mL, pH 8) were incubated in a mixer at 150 rpm and 288–298 K. Small volume of aqueous samples was extracted at pre-set time intervals. The lysozyme concentration in the supernatant was measured after centrifugation while mass balance equations were used to examine the lysozyme concentration adsorbed by dyed nanofiber.

A total amount of 0.03 g nanofiber and variation of lysozyme concentrations in 20 mM sodium phosphate buffer (pH 8) were used to study the batch equilibrium studies of protein adsorption. The solution was then mixed at 150 rpm at 288–318 K for 24 h. 1 mL of samples was collected from the solution, and the lysozyme concentrations in liquid phase, that is supernatant, and the solid phase, which is nanofiber were examined. The effect of salt concentrations, NaCl (0.1–1.0 M) on the elution efficiency of adsorbed lysozyme from the dyed nanofiber membrane was investigated. All experiments were triplicated with <5% standard deviation. The presented data are the mean values examined from each experiment.

### 2.5. Lysozyme Desorption Study 

A total amount of 0.03 g dyed nanofiber was adsorbed by lysozyme through incubating it for 180 min in a 10 mL lysozyme solution (pH 8, 20 mM of Na_2_HPO_4_ buffer) at stirring speed of 100 rpm. After that, buffer was used to rinse away the unbound lysozyme molecules on the nanofiber surface. Then, 10 mL of eluant was poured into the flask and the membrane was incubated for 1 h at stirring speed of 100 rpm. The aliquots of sample were extracted during pre-set time intervals to examine the lysozyme concentration desorbed from dyed membrane. Desorption yield of lysozyme was calculated by dividing the amount of desorbed lysozyme with the amount of lysozyme on the membrane. 

### 2.6. Adsorption of Lysozyme in Flow Process

A flow system was made of membrane column (~0.03 g, with effective area of 3.70 cm^2^), a pump and a water-jacketed reservoir. The lysozyme solution was loaded into the flow system, and 0.5 mL/min of flow rate was maintained (i.e., flux 0.0135 cm^2^/min). A total loading volume of 20 mL lysozyme samples was collected at specific intervals. 

### 2.7. Continuous Removal of Lysozyme in Flow Process

Dynamic binding behavior of lysozyme on the membranes was investigated using an AKTA Prime system (GE Healthcare Biosciences) with a special-designed holder which has an effective area of 3.7 cm^2^ [25]. A volume of 20 mL lysozyme solution was loaded into the holder using super-loop (0.1, 0.5, and 1.0 mL/min; flux 0.027, 0.135, and 0.27 mL/cm^2^ min). Parameters such as loading solution pH and lysozyme concentrations were conducted and studied as followed.

The dynamic breakthrough curve was analyzed in line with the outlet concentrations of lysozyme. The effects of solution pH (pH 7–9) and lysozyme concentration (0.5, 1.0, and 2.0 mg/mL) on the breakthrough curves were examined. At every 1mL, the permeate solution was collected. Lysozyme concentrations of every collected fraction after the breakthrough curve measurement were recorded and calculated.

The permeation flux, *J* (mL/cm^2^·min) in the flow process was calculated using Equation (1) [26,27]:

(1) $$J=\left(\frac{V}{A\cdot t}\right)$$

where *t* represents the operation time (min), *A* represents nanofiber membrane’s effective area (cm^2^), *V* represents the total volume of permeated solution (mL).

The residence time in the membrane, τ (min) was determined using Equation (2) [27,28]:

(2) $$\tau =\frac{\varepsilon \times V_{M}}{F}$$

where *V_M_* represents the membrane volume (~3.39 × 10^−2^ mL), *F* represents the flow rate (0.1–1.0 mL/min) and *ε* represents the porosity of the membrane (~83.45%).

### 2.8. Kinetic Models

Data were fitted into 4 kinetic models to investigate the controlling mechanism and the rate constant of adsorption. Protein adsorption on the nanofiber was described by pseudo-first-order Equation (3) and pseudo-second-order Equation (4), Elovich Equation (5) and intraparticle diffusion Equation (6) kinetic models [19,20,21]. This adsorption mechanism of the first and kinetic models indicates the lysozyme transport to the external surface of the membrane and the surface reaction are rate-controlling steps, respectively.

The linearized pseudo-first-order, pseudo-second-order, Elovich, and intraparticle kinetic rates are as follows:

(3) $$\ln\left(q_{1}{-\;q}_{t}\right)=\ln q_{1}{-\;k}_{1}t$$

(4) $$\left(\frac{t}{q_{t}}\right)=\left(\frac{1}{k_{2}q_{2}^{2}}\right)-\left(\frac{1}{q_{2}}\right)t$$

(5) $$q_{t}=\frac{1}{\beta }ln\left(\alpha \beta \right)+\frac{1}{\beta }ln\left(t\right)$$

(6) $$q_{t}=k_{i}\times t^{0.5}+I$$

where the binding capacity of dye at any given time, *t* (mg/g) was *q_t_*, *q_1_*_,_ *q_2_*; the pseudo-first- and -second-order kinetics rate constants were *k_1_* and *k_2_*, respectively, while the Elovich kinetic constants were *α* and *β*; the intra-particle diffusion rate constant was *k_i_* (mg/g·min^0.5^); lastly, *t* was adsorption time when the system was at equilibrium.

### 2.9. Equilibrium Isotherm Models

The data of lysozyme adsorption by dyed nanofiber membrane were fitted with the equilibrium isotherm models: Langmuir Equation (7), Freundlich Equation (8), and Temkin Equation (9) [19,22].

(7) $$\frac{C^{*}}{q^{*}}=\frac{K_{D}}{q_{\max}}+\frac{C^{*}}{q_{\max}}$$

(8) $${\mathrm{lnq}}^{*}{=\mathrm{lnK}}_{F}+n_{F}{\mathrm{lnC}}^{*}$$

(9) $$q^{*}=\frac{RT}{b}ln\left(K_{T}\right)+\frac{RT}{b}ln\left(C^{*}\right)$$

where C^*^ (mg/mL solution) represents equilibrium lysozyme concentration in the aqueous phase, q* (mg/g membrane) is the amount of lysozyme on the membrane solid phase, and *q_max_* represents the maximum lysozyme adsorption capacity of the membrane (mg/g), *K_D_* represents the Langmuir dissociation constant (mg lysozyme/mL solution). *K_T_* is equilibrium binding constant (mL/g), *n_F_* and *K_F_* are the Freundlich constants related to adsorption intensity and adsorption capacity, respectively. *K_LF_* is the affinity constant for adsorption; *b* is related to heat of adsorption (J/mol), *T* is the temperature (K), and *R* is gas constant (8.314 J/mol·K). 

### 2.10. Dynamic Binding Capacity

Dynamic binding capacity (*DBC*) represents the adsorption efficiency of membrane (lysozyme binding capacity) at defined breakthrough point (*DBC_10%_*). *DBC* of membrane was obtained using Equation (10) [27,28,29,30,31,32,33]. The breakthrough curve revealed the adsorption capacity of the membrane and the amount of lysozyme removal.

(10) $$DBC_{x\%}=\frac{C_{o}\left(V_{x}-V_{d}-\int_{V_{o}}^{V_{x}}xdV\right)}{W_{M}}$$

where *V_x%_* is the applied volume (mL) at *x*% of breakthrough points (*x* = 10), *V_d_* (~1.0 mL) is the volume of residue (mL). The adsorption performance was determined by varying loading solution pH, concentration of lysozyme, and applied flow rate. 

The removal efficacy (*RE*, %) of lysozyme at 10% breakthrough point (corresponding to the ratio of the total amount of lysozyme binding on the membrane at 10% breakthrough to the total amount of feed lysozyme) was calculated according to the following equation.

(11) $$RE\left(\%\right)=\left(\frac{DBC\times W_{M}}{C_{o\times V_{L}}}\right)\times 100$$

where *C_o_* is the initial concentration of lysozyme (mg/mL), *V_L_* is the loading volume of lysozyme solution (7.5 mL in this work), and *W_M_* is the mass of dyed nanofiber membrane (g). 

### 2.11. Dynamic Adsorption Performance Indicator

The membrane adsorber exhaustion rate (*MAER*) for a given membrane bed and the number of bed volumes (*BV*) processed prior achieving 10% of breakthrough point were directly related to the adsorption performance [34,35]. The *MAER* can be calculated using the following equation:

(12) $$MAER=\frac{W_{M}}{V_{b}}$$

where *W_M_* represents the membrane mass (g) and *V_b_* represents the treated volume of lysozyme solution at breakthrough point (mL). In addition, the *BV* can be calculated using the following equation:

(13) $$BV=\frac{V_{b}}{V_{M}}$$

where *VM* represents the total volume of membrane bed (mL). 

Moreover, the mass transfer zone (*MTZ*) was defined as the region in which the adsorption of lysozyme occurs between 10% and 50% of the inlet concentration of lysozyme. The adsorption efficiency of membrane bed is influenced by the existence of mass transfer zone, and the membrane is preferrable to have thinnest mass transfer zone. *MTZ* can be calculated using following equation [36,37,38,39]:

(14) $$MTZ=Z\left(1-\frac{t_{10\%}}{t_{50\%}}\right)$$

where *t_10%_* and *t_50%_* are the time when the lysozyme concentrations at the outlet equal to 10% and 50% inlet concentration of lysozyme, and *Z* is the height of the entire adsorption bed (cm), respectively (min).

## 3. Results and Discussion

### 3.1. Physical and Chemical Properties of the Nanofibers

**Figure 2:**
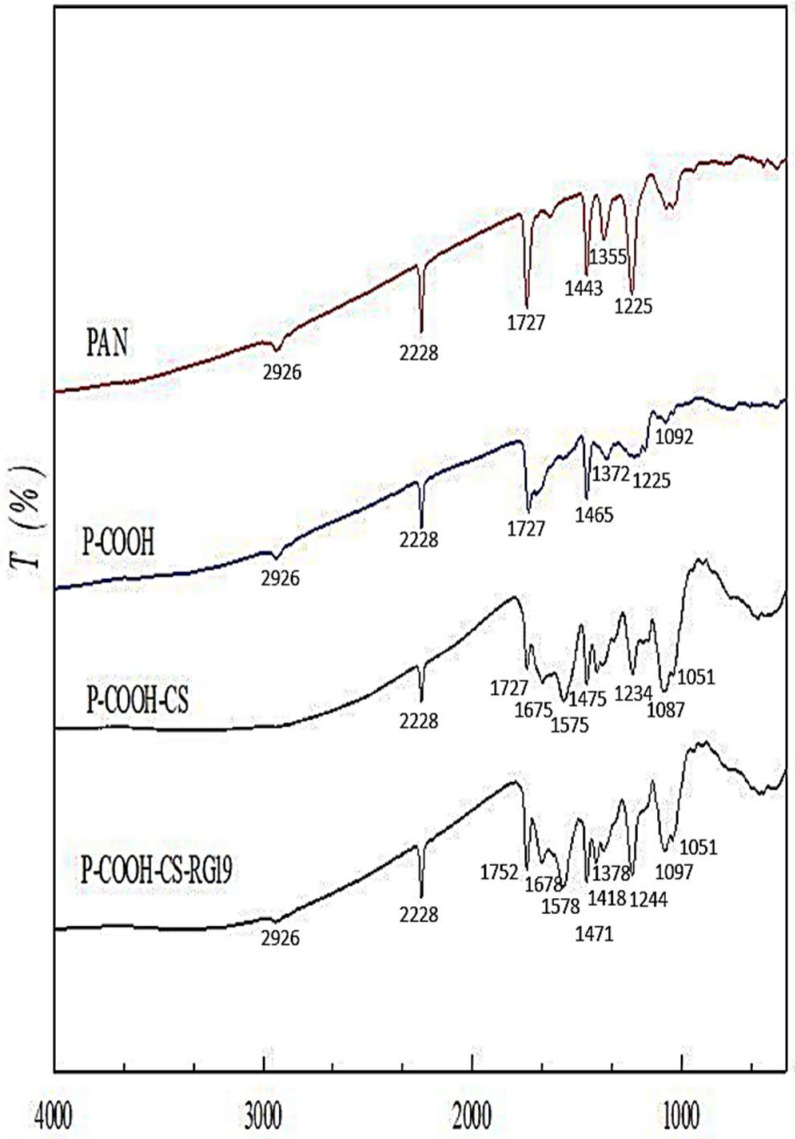
FTIR spectra of (**a**) PAN, (**b**) P-COOH, (**c**) P-COOH-CS, (**d**) P-COOH-CS-RG19.

The results of the FTIR analysis of the each-stage membranes prepared (PAN, P-COOH, P-COOH-CS, and P-COOH-CS-Dye) have been discussed in a previously published paper [16]. Based on the analysis of FTIR spectra, the functionalized membranes prepared at each stage have specific functional groups, and the absorption peaks of these membranes produced differentiated spectra, indicating that the modification of the membranes at each step is successful. As reported in the previously published paper, the peaks at 1225 cm^−1^ and 2926cm^−1^ were −CH_2_ functional groups, and 2228 cm^−1^ represented −CN functional group can be found in most of the membranes, showing the modified membranes have not been critically destroyed after the alkali treatment and dye immobilization. The peak of 1727 cm^−1^, represent the C=O stretching which can be observed in the unmodified membrane and the membranes without dye immobilization. As for the membrane coupled with CS, the presence of CS was indicated by several peaks, such as 1675 cm^−1^ and 1678 cm^−1^ representing the amide I band, 1575 cm^−1^ and 1574 cm^−1^ representing −NH_2_ functional groups and 1033–1142 cm^−1^ represent C-N functional groups, as a functional group of amines on the membranes. The amino group, −NH_2_, was observed at the peaks of 1575 cm^−1^ and 1578 cm^−1^ on both membranes coupled with CS, indicating there are amine residues in the CS coupled on the membranes. The peaks of 1678 cm^−1^ and 1675 cm^−1^ were strong carbonyl bands which were probably caused by amide or carboxylic acid. As for P-COOH-CS-RG19, the functional group of −SO_3_^−^ could be indicated by the peak of 1051 cm^−1^, which was S=O stretching falling in the range of 1070–1030 cm^−1^. The resultant peak indicated that the dye molecules have firmly attached to the CS coupled nanofiber membranes. The FTIR spectra for each stage membrane prepared were shown in Figure 2.

**Figure 3:**
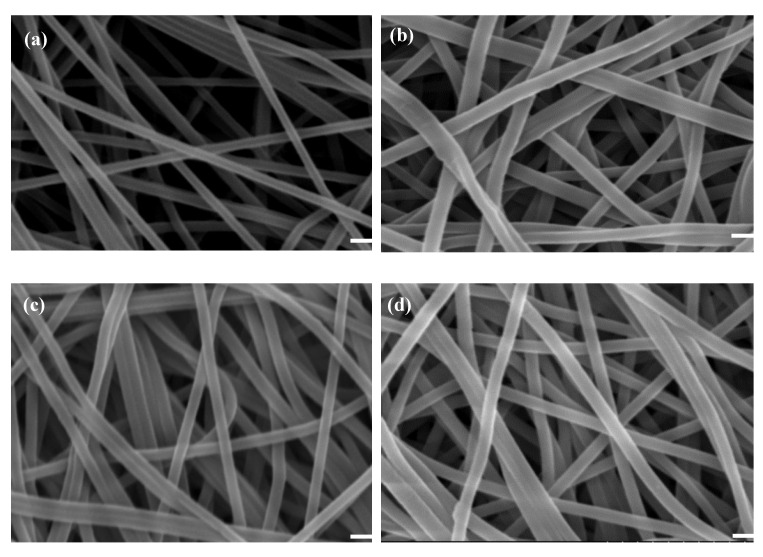
SEM images of (**a**) PAN, (**b**) P-COOH, (**c**) P-COOH-CS, and (**d**) P-COOH-CS-RG19. Bar scale: 1000 nm.

The surface morphological attributes of the nanofibers are displayed in Figure 3a–d, with the diameter ranging from 300–600 nm, respectively. The results showed that the PAN nanofiber’s morphology is neat and tubular structures. The thickness of PAN nanofibers ranged from 347–458 nm as observed from the displayed SEM images, which was comparatively narrower than the other types of nanofibers. Besides the thickness, most of the tubular structures of PAN nanofibers were also observed to look straight. After the PAN nanofiber was hydrolysed, the morphology of P-COOH nanofibers contained some degree of twist. The thickness of P-COOH nanofibers was increased after hydrolysis and it ranged from 337–728 nm as displayed. After the chitosan and dye molecules are coupled with the P-COOH nanofiber membrane sequentially, no significant alteration of the morphology of the nanofibers produced was observed. 

**Figure 4:**
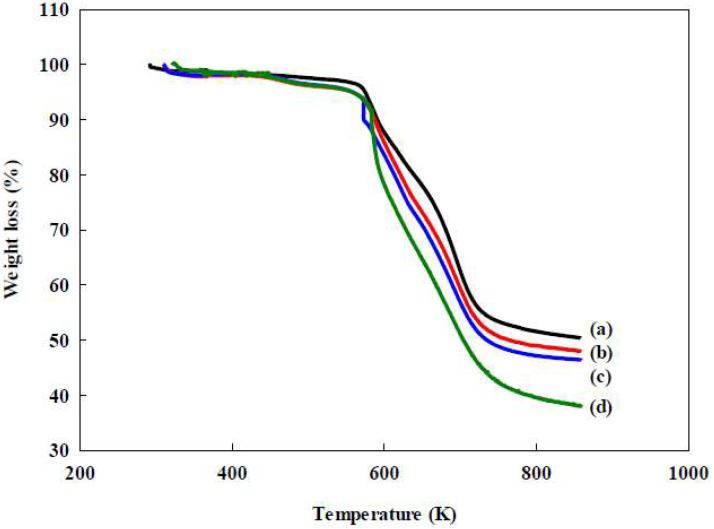
TGA curves of (**a**) PAN, (**b**) P-COOH, (**c**) P-COOH-CS, and (**d**) P-COOH-CS-RG19.

Similar results for various nanofibers by TGA analysis were also recorded in our previous studies [13,14,15,16], such as PAN, P-COOH, P-COOH-CS, P-COOH-CS-RO4 [15], and P-COOH-CS-RB49 [16]. The results indicated that the TGA analysis under different dyes immobilized onto the modified chitosan nanofiber [e.g., Reactive Orange 4 (RO4), Reactive Blue 49 (RB49), and Reactive Green 19 (RG19) dyes] has very similar curves. The order of stability of nanofiber membrane by TGA analysis was PAN > P-COOH > P-COOH-CS > P-COOH-CS-RG19 as shown in Figure 4 [15]. 

The highest stability of nanofiber membrane is the unmodified PAN membrane while P-COOH-CS-RG19 showed lower stability than P-COOH-CS and P-COOH. The thermal stability of the membrane reduced as it went through a series of processes, which is in agreement with the results observed by Show et al. (2020) [16] The nanofiber membrane immobilized with dye molecules also exhibited more loss compared to others above 700 K, indicating the dye molecules are less thermal recalcitrant. All membranes are quite stable below 573 K, and there was no significant loss of membrane weight. All membranes exhibited a similar trend below 573 K, indicating similar thermal stability as they contained the same major component, PAN, in their structures [15]. The weight loss of these membranes was approximately the same value below 373 K, which might be a result of the loss of moisture content. Hence, the P-COOH-CS-RG19 dyed nanofiber with high thermostability can be applied in the application of biological wastewater treatment.

FTIR results provide qualitative insights into these nanofibers (Figure 2). To quantify the functional groups (e.g., −COOH and −NH_2_) on the nanofiber membranes, the measurement was performed based on the adsorption of the TBO and AO7, for carboxyl and amine groups, respectively. In this study, the molecular weight of CS coupled with the P-COOH nanofiber membrane utilized in the preparation of the P-COOH-CS nanofiber membrane was 50 kDa, 210 kDa, and 340 kDa. The amine (−NH_2_) and carboxylic (−COOH) concentrations on the P-COOH, and P-COOH-CS nanofibers are summarised in Table 1. The content of carbonyl groups on the P-COOH nanofiber membrane was 266.66 μmol/g. The content of residual amine groups on the P-COOH-CS was 179.06 μmol/g, 145.39 μmol/g, and 112.65 μmol/g for 340 kDa, 210 kDa, and 50 kDa CS, respectively. This is because the higher the molecular weight of CS, the more the functional groups of amines on the membrane, resulting in a higher coupling amount. However, the coupling efficiency would be higher with a low molecular weight of CS. Hence, the different molecular weights of the CS used in the coupling reactions significantly affected the adsorption performance of dye and lysozyme as described in the following section. 

### 3.2. Operating Parameters for the Adsorption of Lysozyme

#### 3.2.1. Coupling with Different Molecular Weights and pH of Chitosan

**Figure 5:**
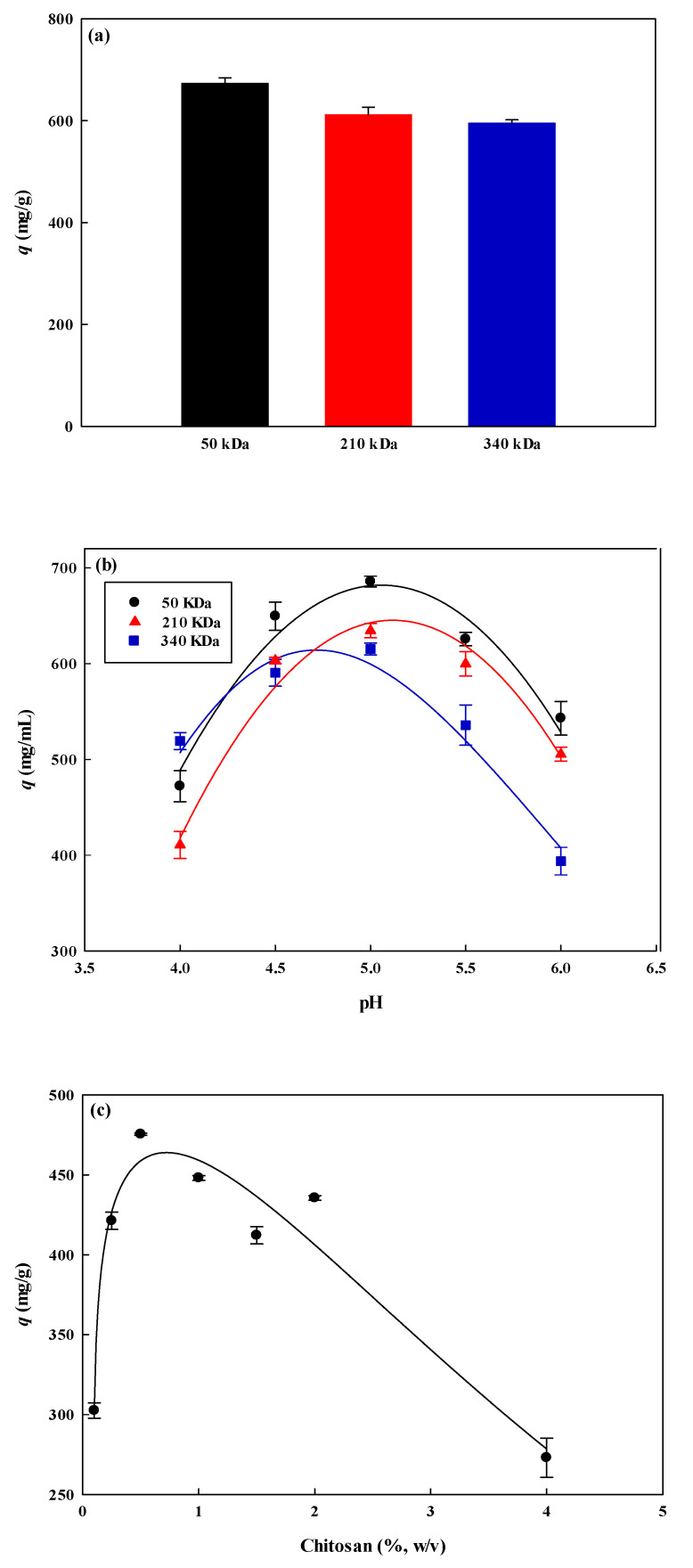
The effect of (**a**) molecular weight of chitosan, (**b**) solution pH, and (**c**) concentration of chitosan (CS) coupled with P-COOH nanofiber membrane on the adsorption capacity for lysozyme.

In the experiment, three different molecular weights of CS (50, 210, and 340 kDa) were used and their effects on the adsorption capacity of lysozyme were investigated. These different molecular weights are studied to determine the effect of the molecular weights of CS on the adsorption capacity of lysozyme by the P-COOH-CS-RG19 membrane. These molecular weights are chosen for the investigation using different ranges of molecular weight of CS coupling in nanofiber membrane, as 50–190 kDa is considered as low molecular weight CS, 190–310 kDa is medium molecular weight CS and those above 310 kDa is high molecular weight CS [40,41,42]. A 3 mg/mL dye concentration was used to immobilize on the chitosan-modified membrane. It was carried out with a 2.0 mg/mL lysozyme solution at an adsorption pH of 9. The Figure 5a showed that the adsorption capacity of lysozyme by the P-COOH-CS-RG19 nanofiber membrane decreased with the increasing molecular weight of chitosan. The usage of 50 kDa, 210 kDa, and 340 kDa of chitosan molecular weight have obtained the following adsorption capacity for lysozyme: 686.69 mg/g, 630.89 mg/g, and 612.29 mg/g, respectively. This may be due to the different contents of amine groups on the CS modified nanofiber, resulting in the concentration of immobilized dyes being affected accordingly. As reported, CS contains the amine group as one of the reactive functional groups which cause the CS molecules to couple with the RG19 dye molecules [12]. It was found that the order of the content of amine groups on the immobilized chitosan nanofiber was 50 kDa > 210 kDa > 340 kDa as shown in Table 1. Therefore, chitosan with a molecular weight of 50 kDa was selected as the optimal molecular weight for subsequent experiments. Based on the experimental results, this may be due to the complex and random framework easily formed by the dye molecules and the higher molecular weight chitosan macromolecules on the membrane, resulting in lysozyme molecules not being easily accessible. The effects of coupling pH of CS with P-COOH nanofiber membrane on the adsorption capacity of lysozyme onto the dyed nanofiber membrane were further investigated and the results are presented in Figure 5b. As the coupling pH of CS (50 kDa) increased from 4 to 6, the adsorption capacity for lysozyme was drastically affected by the coupling pH. It was reported that the adsorption behaviour of protein is affected by the change of pH as pH is capable to alter the surface charge of both membrane and protein surfaces [15]. In this case, pH 5 was the optimal pH to couple CS on the nanofiber membrane.

#### 3.2.2. Coupling Concentration of Chitosan

The CS-modified membranes with high amine groups can be not only a weak cation (−NH_2_) but also a weak anion (−NH_3_^+^) exchange membrane. According to the isoelectric point of the lysozyme (*pI* 11.0) [43], it can be used to predict whether the nanofiber membrane has an adsorption reaction to the lysozyme molecule. In this case, the membrane adsorption process is affected by the flexibility and size of the lysozyme molecule, the diffusion behaviour in the bulk solution, and nanofiber membrane properties (e.g., porosity, nanofiber diameter, and hydrophilicity). In this study, the effect of P-COOH membrane-conjugated CS concentration from 0.1 to 4.0%, *w*/*v* on the adsorption capacity of lysozyme was investigated. As mentioned above, the optimal pH for the coupling of CS molecules was 5.0. The residual amine groups of the activated P-COOH-CS can be simply modified with the reactive RG19 dye molecules after coupling reaction. Figure 5c has displayed the effect of coupling different concentrations of CS to the P-COOH membrane on lysozyme adsorption. For P-COOH-CS-RG19, it was found that the adsorption capacity of P-COOH-RG-RG19 for lysozyme was 302.52 mg/g at a mass concentration of 0.1% CS. The maximum adsorption capacity was approximately 475.45 mg/g at a mass concentration of 0.5% CS. However, at above 0.5%, the binding capacity drops significantly to 273.05 mg/g at a mass concentration of 4.0% CS. Based on these results, CS molecular weight, concentration, and pH would seriously affect the adsorption capacity of lysozyme, these parameters also significantly affect the immobilization density of dye molecules. Hence, the adsorption capacity for lysozyme is also affected. The optimal conditions for coupling CS with P-COOH nanofiber membrane are CS molecular weight of 50 kDa, coupling pH of 5.0, and CS concentration of 0.5%.

### 3.3. Immobilization Parameters of Dye

#### 3.3.1. Immobilization pH

**Figure 6:**
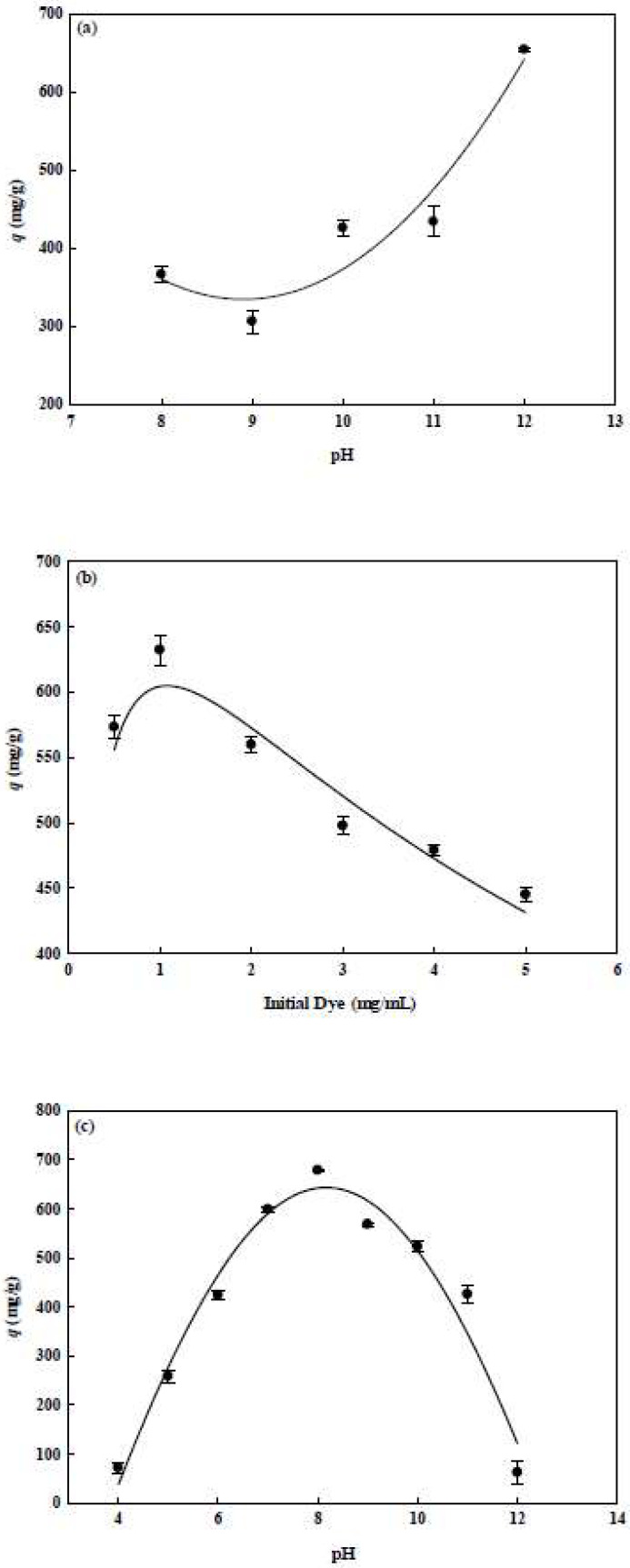
The effect of (**a**) dye solution pH, (**b**) initial dye concentration, and (**c**) lysozyme adsorption pH on the adsorption capacity for lysozyme.

The dye molecules can couple to the CS-modified nanofiber membrane; while under alkaline condition, the reactive chlorine displacement occurs due to molecular nucleophilic substitution [14,16]. Nucleophilic reactions are easily generated by the high pH, which promotes the nucleophilic reaction with the amine or hydroxyl groups on the CS modified nanofiber. Hence, dye molecules are easily immobilized onto the CS-modified nanofiber. In this work, one step was adopted to perform the dye immobilization on the CS modified nanofiber membrane, using a solution with high salt and high pH, at 70 °C of reaction temperature for 3 h. Adjusting the pH value of the immobilized dyes in alkaline conditions, the effect of immobilization pH of dye on the adsorption capacity for lysozyme was investigated. The experiment was carried out using the dye solutions at various pH values from 8 to 12 and the concentration of the lysozyme protein solution was 2.0 mg/mL at pH 7. Generally, P-COOH-CS-RG19 nanofiber increased as the pH of the dye solution increased, there was a minimum adsorption capacity (286.43 mg/g) for lysozyme at pH 9, and the maximum adsorption capacity for lysozyme at pH 12 was 654.35 mg/g as shown in Figure 6a. Therefore, the pH 12 of dye solution was selected as the optimum condition for dye immobilization. The results showed that the higher the density of the immobilized dyes obtained at a higher pH, resulting in higher adsorption capacity for lysozyme. This statement is supported by the study of Ng et al. (2019) [15], where higher density of immobilized dye molecules led to exponential increase of lysozyme adsorption by the membrane to a maximum limit, for example maximum adsorption of lysozyme to 369.92 mg/g. However, the study reported that a steric barrier might occurred using 111.49 µmol/g dye concentration due to the reduced possibility of interaction between functional groups of dye molecules with lysozyme [15].

#### 3.3.2. Initial Dye Concentration

The immobilized RG19 dye density on the nanofiber membranes is another important parameter influencing the adsorption performance of proteins [16]. Figure 6b has illustrated the relationship between the adsorption capacity for lysozyme and the dye initial concentration (0.5–5 mg/mL). The variation of adsorption performance by nanofiber membranes might resulted by the molecular mass of immobilized dyes, charge distribution and arrangements of dye molecules as well as the interactions between lysozyme and dye molecules (e.g., ionic, hydrophobic, or specific interactions) [14,16]. 

As shown in Figure 6b, when the maximum adsorption capacity for lysozyme on the P-CS-Dye was 632.43 mg/g at 1.0 mg/mL dye concentration, the corresponding immobilized dye mass was 384.45 mg RG19/g membrane (0.271 mol RG19/g). Therefore, it is recommended that the optimal initial dye concentration selected was used for the immobilization of RG19 on the P-CS-RG 19 nanofiber membrane. An increase in dye loading (>1.0 mg/mL) resulted in a significant decrease in adsorption capacity. This reduction of adsorption capacity for lysozyme may resulted by the steric effect of the high amount of dye molecules on the P-CS-Dye nanofiber. Therefore, the dye molecules not able to easily access with the active binding site of lysozyme molecules. This explains that the degree of steric hindrance between the dye and lysozyme molecules on the membrane is a significant factor in the binding capacity.

### 3.4. Lysozyme Adsorption by Dyed Nanofiber Membrane

#### 3.4.1. Adsorption pH

Studies of the adsorption of lysozyme by dyed membranes were carried out in a pH range of 4–12. As observed in Figure 6c, the adsorption pH values have a great impact on the binding capacity of dyed membranes. The maximum adsorption capacity of lysozyme for P-COOH-CS-RG19 was pH 8, which was 678.66 mg/g. However, lower binding capacities was observed in dyed nanofiber membrane for the pH values below pH 7 and above pH 8. The positive charge density of lysozyme molecule rises with lower pH values while dye molecules carried negative charges. An asymmetric distribution of the surface charge on the lysozyme molecule was assumed as indicated by the tendency of adsorption capacity. As such, the positively charged amino groups may focus on a region of lysozyme molecule only even if more amino groups were existed on lysozyme molecules at low pH values; where similar results have reported by several studies [14,15]. However, the net charge concept could not be used to explain the adsorption capacity of protein satisfactorily. Likewise, the adsorption behaviour may not be controlled by the charge distribution on the lysozyme molecule [14,15]. As the adsorption pH increased from pH 8 to 11 (near the *pI* value of lysozyme), the adsorption capacity decreased to 426.09 mg/g for P-COOH-CS-RG19, respectively. The reason may be that the number of lysozyme molecules protonated has less positively charged groups at pH 11. This phenomenon contributes to a weaker electrostatic effect on positively charged lysozyme and negatively charged dyed membranes. When the adsorption was conducted at pH 4 and 12, the adsorption capacity for the dyed nanofiber membrane was significantly reduced to 71.86 and 62.52 mg/g, respectively. 

Based on the experimental results, it should be noted that the isoelectric point of lysozyme is 11.0 [43] and it tends to be cationic at the adsorption pH values below than isoelectric point. Also, the *pKa* value of the sulfonate groups (−SO_3_^−^) of the Reactive Green 19 dye is 0.8 [44] and its charge is negative under the operating conditions. When considering the charge of the species, it can be concluded that electrostatic interactions were the main driven force for the interaction between the dye molecule and the lysozyme molecule. However, in order to explain the optimal adsorption pH of the presented study, it may not use the electrostatic interactions alone to explain this adsorption behaviour. It should be considered the surface characteristic of lysozyme and the three-dimensional orientation of the lysozyme. Moreover, the interaction between the dye and the lysozyme molecules may be contributed by the combination of electrostatic, hydrophobic, and van der Waals forces [44]. In conclusion, the optimal pH for the lysozyme adsorption was pH 8 for the P-COOH-CS-RG19 membrane in a batch mode. Therefore, pH 8 was used in following experiments.

#### 3.4.2. Kinetic Study

**Figure 7:**
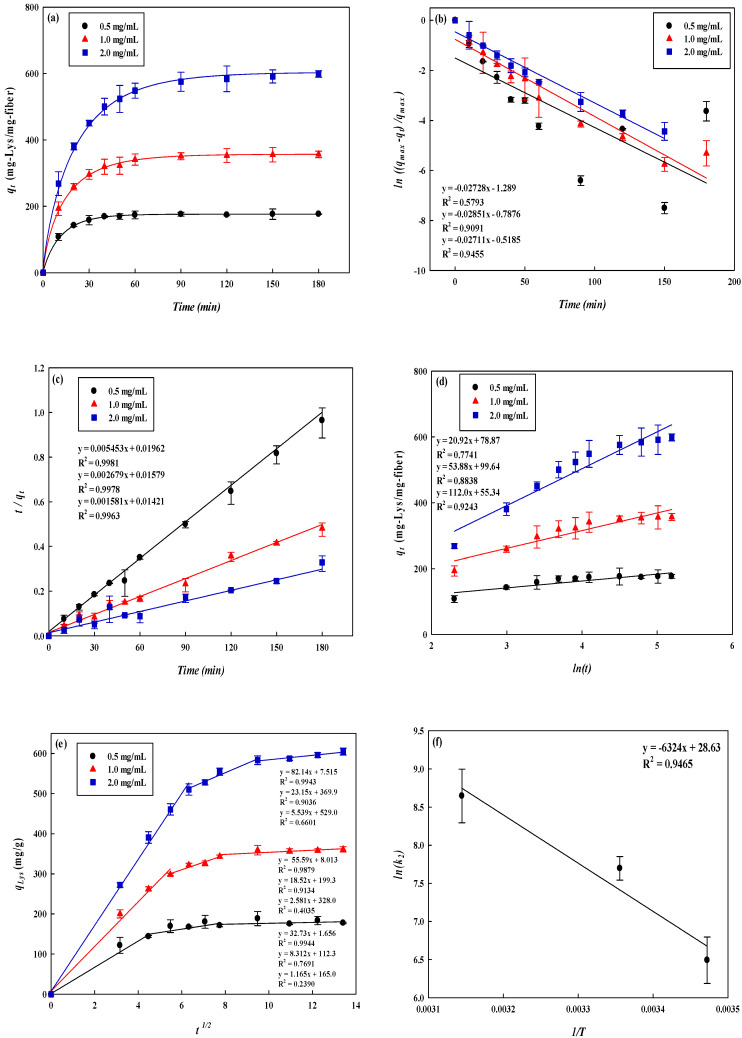
(**a**) Adsorption rates for lysozyme by P-COOH-CS-RG19. Kinetic adsorption of lysozyme fitted by (**b**) pseudo first-order model, (**c**) pseudo-second-order model, and (**d**) Intraparticle diffusion model. (**e**) membrane surface diffusion and intra-particle (membrane) diffusion. (**f**) the activation energy for the adsorption of lysozyme.

Figure 7a and Table 2 show the results of the kinetics of lysozyme removal by P-COOH-CS-RG19 at lysozyme concentration ranges from 0.5–2.0 mg/mL. It was found that the removal of lysozyme approached to equilibrium after about 60 min. It indicated that P-COOH-CS-RG19 has a high removal rate for lysozyme. When the initial lysozyme concentration increased from 0.5 to 2.0 mg/mL, the experimental results showed that the removal of the lysozyme increased from 176.35 mg/g to 598.66 mg/g. Higher concentration of adsorbed lysozyme allowed better removal rate due to the concentration difference between solid and liquid phases. The drastic concentration difference has facilitated the collision of lysozyme with the membrane binding sites. As the negatively charged dye molecules are immobilized on the surface of the nanofiber membrane, the positively charges lysozyme molecules are adsorbed at the outer surface of dyed membrane. The adsorption models: pseudo-first-order, pseudo-second-order, Elovich, and intraparticle diffusion kinetic models (see Equations (3)–(6)), were applied in the kinetic studies for the removal of lysozyme on the dyed nanofiber membrane and determining the removal rate constant for lysozyme adsorption.

The removal rate constants derived from the fitting of the pseudo-first- and second-order kinetic adsorption model, as well as the P-COOH-CS-CEW adsorption capacity for lysozyme are tabulated in Table 2. The pseudo-first-order kinetic and Elovich models showed the low *R^2^* values, 0.58–0.94 and 0.77–0.92 in Figure 7b,d, respectively; therefore, these kinetic models are not appropriate for the description of the adsorption behavior. Figure 7c shows the performance of the data fitting using pseudo-second-order kinetic model, that a high *R^2^* values (>0.99) was obtained, showing the promising suitability of pseudo-second-order- kinetic model in describing the lysozyme adsorption behavior. Furthermore, the surface reaction on the membrane may be the rate-determining step of lysozyme adsorption. Using 0.5, 1.0, and 2.0 mg/mL of lysozyme concentration, the obtained lysozyme adsorption capacity (*q_cal_*) is 185.60, 367.69 and 610.90 mg/g, respectively. It is noteworthy that these values are quite close to the maximum amount of the adsorbed lysozyme (*q_exp_*) by P-COOH-CS-RG19 and the activation energy for the adsorption of lysozyme obtained from Figure 7f was 5.26 × 10^4^ J/mol as shown in Table 2. After the lysozyme was adsorbed on the surface of the adsorber membrane surface, the diffusion rate may vary with the influence of factors such as membrane porosity and pore size, and the diffusion model. It can be divided into membrane surface diffusion and intra-particle (membrane) diffusion. As shown in Figure 7e, it was observed that when the lysozyme concentration is between 0.5 mg/mL and 2.0 mg/mL, the immobilized dye membrane for adsorption of lysozyme has a three-stage diffusion. If the rate-limiting step is intraparticle diffusion, a plot of adsorption capacity against the square root of the contact time should yield a straight line passing through the origin [45,46,47]. The intraparticle diffusion plots show multi-linearity in the biosorption process indicating that three steps are operational. The first, sharper stage can be attributed to the diffusion of lysozyme through the solution to the external surface of the membrane or the boundary layer diffusion of the lysozyme. The second stage describes the gradual adsorption, where intraparticle diffusion is rate-limiting and the third stage is attributed to the final equilibrium for which the intraparticle diffusion starts to slow down due to extremely low lysozyme concentration left in the solution. The three stages in the plot suggest that the adsorption process occurs by surface adsorption and intraparticle diffusion (microporous). The values of the intraparticle diffusion rate constant, *k_i_*, calculated are shown in Table 3. The results indicate that the intraparticle diffusion rate increases with increasing initial lysozyme concentration in solution. An increase in the initial concentration of lysozyme yields a higher concentration gradient which eventually leads to faster diffusion and rapid adsorption. 

#### 3.4.3. Thermodynamic Study

**Figure 8:**
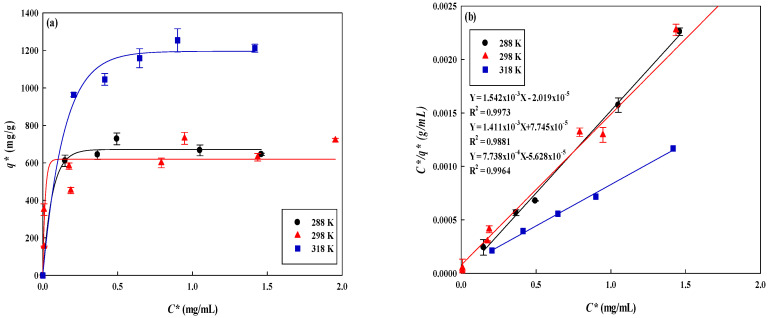

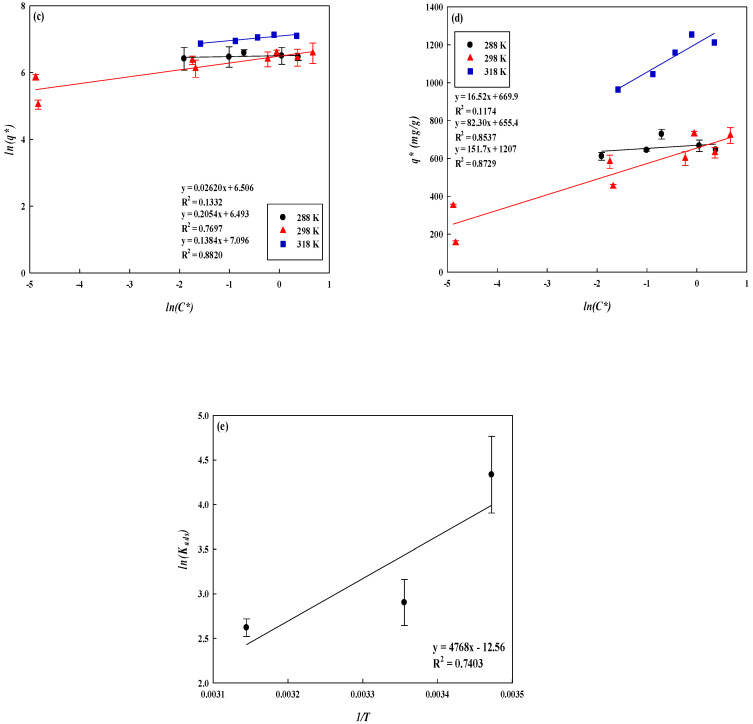
(**a**) Effect of temperature on the equilibrium isotherm curves for the adsorption of lysozyme on P-COOH-CS-RG19 nanofiber membrane, (**b**) Langmuir model plot of C*/q* against C*, (**c**) Freundlich model plot of ln(q*) against ln(C*), (**d**) Temkin model plot of q* against ln(C*), and (**e**) Van ’t Hoff plot for the adsorption of lysozyme on P-COOH-CS-RG19 nanofiber membrane.

In this experiment, the change of the adsorption capacity of the membrane for different concentrations of lysozyme (0.0−5.0 mg/mL) at temperatures range from 288–318 K was investigated to obtain the maximum adsorption capacity and adsorption constant of the dyed membrane, thermodynamic parameters (Δ*G^o^*, Δ*H^o^*, and Δ*S^o^*). The results are shown in Figure 8a. It was found that the adsorption capacity for lysozyme increased with increasing the operating temperatures. When the temperature is at 288 K, 298 K, and 318 K, the adsorption capacity is 648.51 mg/g, 708.71 mg/g, and 1293.66 mg/g, respectively. At 298 K and 298 K, the adsorption equilibrium was reached when the lysozyme concentration was about 3.0 mg/mL, while the temperature of 318 K reached the adsorption when the lysozyme concentration was about 4.0 mg/mL. The experimental data were fitted using the three adsorption equations of Langmuir, Freundlich, and Temkin (Figure 8b–d). The linear coefficients (*R^2^*) were used to determine the most suitable model to describe the adsorption behaviour as shown in Table 4. Among the isotherm models, the Langmuir model was found to be the most suitable to describe the adsorption for lysozyme. The *K_d_* value determines the affinity of the dyed nanofiber to the lysozyme. The reciprocal of the *K_d_* value is the Langmuir adsorption constant (*K_L_*). The *K_L_* value at 288, 298, and 318 K was 76.39, 18.22, and 13.73 mL/mg, respectively. The larger the *K_L_*, the greater the affinity to lysozyme, which is more favourable for the adsorption behaviour. Hence, the lower temperature was more favourable to the affinity interaction between dyed adsorbent and lysozyme. However, in this case, the adsorption capacity for lysozyme was found to be lower at a lower temperature. The thermodynamic parameters were shown in Table 5.

Δ*G^o^* can be calculated from the *K_L_*, and the result shows that Δ*G^o^* < 0 is spontaneous adsorption; plotting ln(*K_L_*) against (*1/T*) as shown in Figure 8e, it can be obtained, Δ*H*^o^ < 0 (corresponding to −3.96 × 10^4^ J/mol). The results showed that it is an exothermic reaction and the higher the temperature, the higher the adsorption capacity, and Δ*S^o^* > 0 means that corresponds to an increase in the degree of freedom of the adsorbed species. the adsorption reaction is favourable.

### 3.5. Lysozyme Elution 

**Figure 9:**
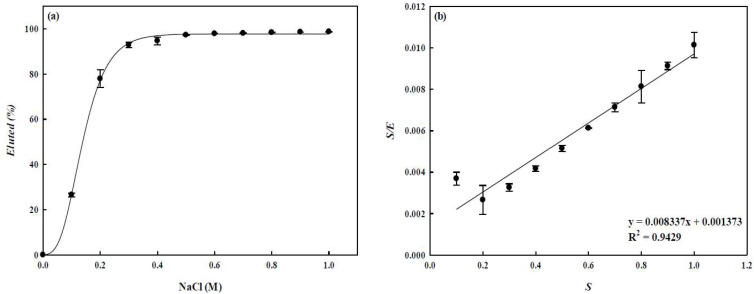
(**a**) Elution efficiency (*E*) for the adsorbed lysozyme from the P-COOH-CS-RG19 nanofiber membrane by a step-wise elution method (i.e., S: 0–1 M NaCl), (**b**) Linear plot of *S/E* against *E*.

In this experiment, the effect of NaCl concentration on the elution efficiency (*E*) was carried out in a batch mode. After the adsorption process, the nanofiber membrane was washed with buffer 3 times, and then the desorption experiments were carried out with different NaCl concentrations (*S*: 0.1–1.0 M) by a stepwise method. The results are shown in Figure 9a and 98.04% of lysozyme can be eluted from the P-COOH-CS-RG19 nanofiber membrane when the 0.7 M NaCl was used for the elution. By using linearized Monod-type equation [48], the maximum amount of lysozyme eluted from the dyed nanofiber and the concentration of NaCl required to elute 50% of adsorbed lysozyme were approximately 100% and 0.17 M NaCl, respectively as shown in Figure 9b. Hence, to completely elute the adsorbed lysozyme, 1 M of NaCl was selected as the optimum concentration of elution solution for the desorption. The results showed that the binding forces of the dyed nanofiber membrane to the lysozyme molecule were mainly ionic interactions.

### 3.6. Continuous Flow Process

**Figure 10:**
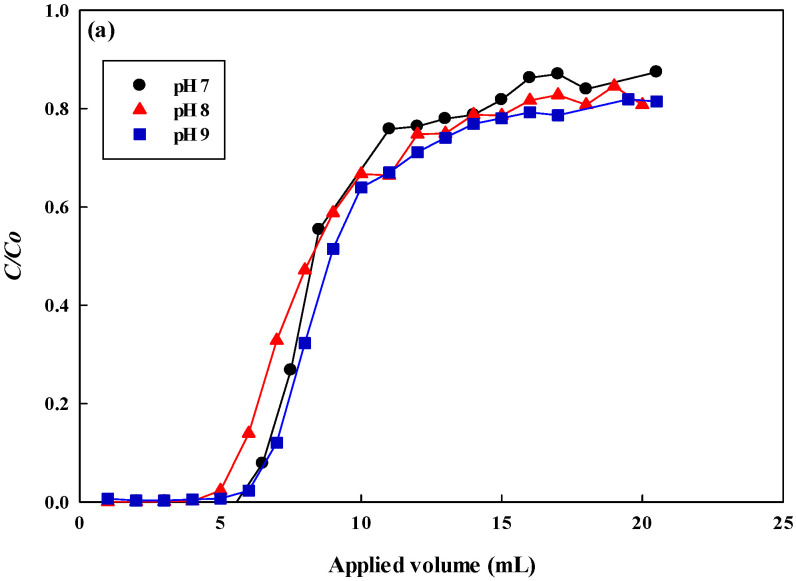

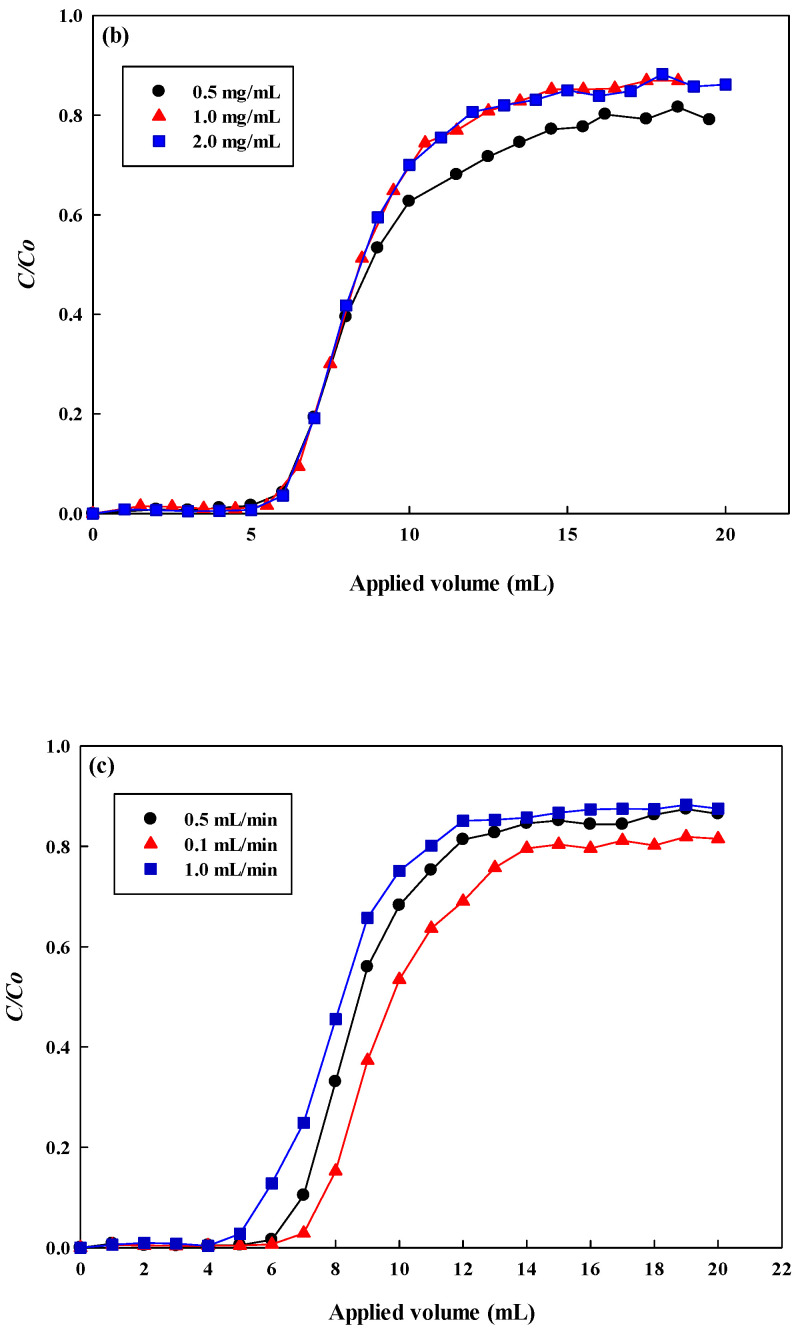
Continuous flow process for the removal of lysozyme by P-COOH-CS-RG19 nanofiber membrane (one piece membrane 0.03 g, effective area 3.7 cm^2^): (**a**) pH (7–9) at a constant lysozyme concentration (2.0 mg/mL) and flow rate (0.5 mL/min), (**b**) lysozyme concentration (0.5–2.0 mg/mL) at a constant pH 9 and flow rate 0.5 mL/min, (**c**) flow rate (0.1–1.0 mL/min) at a constant pH 9 and concentration 2.0 mg/mL.

In a well-mixed batch mode, the maximum adsorption capacity for lysozyme was found to be at pH 8. In this experiment, various pH values (pH 7–9) were further used to evaluate the adsorption performance of breakthrough curves of lysozyme in the continuous membrane chromatography, and the dynamic adsorption was carried out by a newly designed reactor and a fully automatic programmable protein liquid chromatography (GE Healthcare), and the feed volume of lysozyme (2.0 mg/mL) was 20 mL at a liquid flow rate of 0.5 mL/min. Moreover, various lysozyme concentrations (0.5–2.0 mg/mL) and operating flow rates (0.1–1.0 mL/min) were used to find the optimal adsorption conditions for dyed nanofiber membranes. As shown in Figure 10a when the feed lysozyme solution was pH 9, there is a higher performance of the breakthrough curve. The order of dynamic adsorption capacity for lysozyme at 10% breakthrough point was pH 9 (458.27 mg/g) ≥ pH 7 (457.63 mg/g) > pH 8 (380.33 mg/g) (i.e., *C/C_o_* = 0.1). As described in Section 3.4.1, the order of the dynamic and static adsorption performance of lysozyme in-flow and well-mixed systems under different pH values was different. This may be due to the contact time between lysozyme and nanofiber membrane in both systems not being the same, resulting in different adsorption performances. The dynamic binding capacity was also affected by the liquid-liquid mixing and mass transfer zone in a flow system. The experiments with different lysozyme concentrations were carried out with the concentration of lysozyme between 0.5 to 2.0 mg/mL as shown in Figure 10b. In the early stage (*C/C_o_* = 0.1), the adsorption performances for lysozyme were approximately the same (458.27 mg/g). At different operating flow rates as shown in Figure 10c, the dynamic adsorption performance for lysozyme decreased with the increasing flow rate of the feed solution. The dynamic binding capacity at *C/C_o_* for 0.1 mL/min (residence time *τ* = 1.697 s), 0.5 mL/min (residence time *τ* = 0.340 s), and 1.0 mL/min (residence time *τ* = 0.170 s) was approximately 515.32 mg/g, 458.27 mg/g, and 380.33 mg/g, respectively. This is due to the residence time of liquid flow on the membrane being shorter at a higher flow rate, resulting in lower adsorption performance.

**Figure 11:**
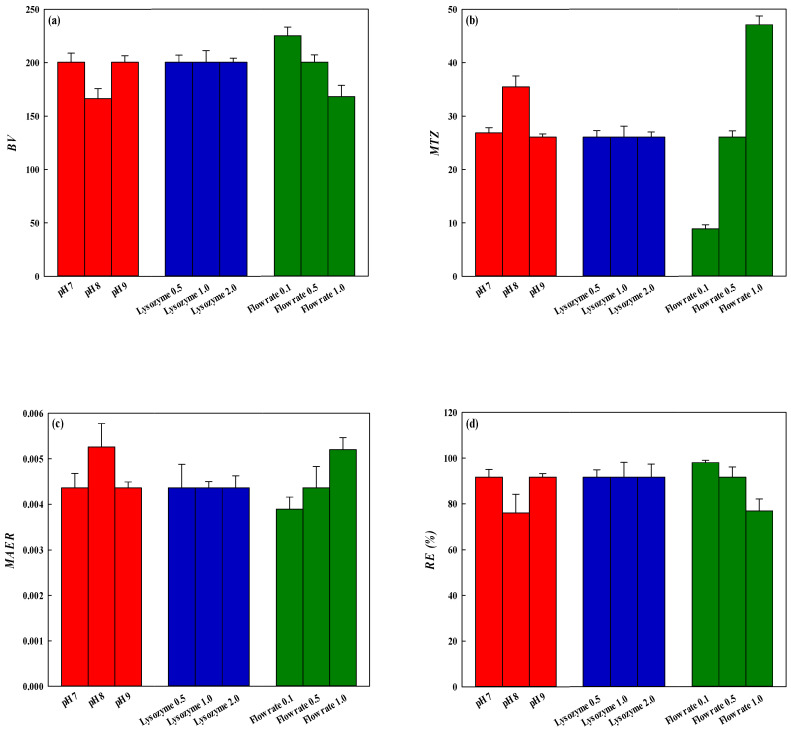
(**a**) *BV*, (**b**) *MTZ*, (**c**) *MEAR*, (**d**) *RE* (%) obtained under different conditions in different flow processes.

The permeation flux in the flow process (mL/cm^2^·min) Equation (1) during the continuous running was found to maintain a constant value. It meant that the dyed membrane was not blocked during the removal process of lysozyme. Moreover, *BV* Equation (12), *MAER* Equation (13), and *MTZ* Equation (14) were also used to analyse the three indicators of dynamic adsorption efficiency and the results are shown in Figure 11a–c. Among them, the larger the *BV* value, the smaller the *MAER* value, and the smaller of *MTZ*, the higher the removal efficiency for lysozyme. The *MTZ* progresses through the regions of membrane adsorption bed, as more lysozymes pass through until lysozyme begins to appear in the effluent. The optimal removal of lysozyme on the dyed nanofiber membrane was the lysozyme concentration of 2.0 mg/mL, adsorption pH of 9, and flow rate of 0.1 mL/min under the operating conditions selected in this work. Based on the 10% breakthrough point as a reference for the volume of lysosomes introduced, the removal efficiency of lysozyme was 95–100% under all operating conditions. However, as the volume of lysozyme introduced was 7.5 mL (corresponding to 219.30 bed volumes for one piece of membrane), the removal efficiency (*RE*) of lysozyme Equation (10) was shown in Figure 11d. At the lysozyme concentration of 2.0 mg/mL and pH 9, operating flow rates of 0.1, 0.5, and 1.0 mL/min, lysozyme removal efficiencies were 100.0%, 91.66%, and 76.89%, respectively.

### 3.7. Remarks of the Applications of the Nanofiber Used in This Work

In the preparation of functionalized nanofiber membranes, the nanofiber membrane prepared at each stage in this work has its functionalities and applications, such as the PAN-based nanofibers for use in various fields [49], the P-COOH nanofiber for use in lysozyme purification [50], removal of waste dye, and metal [27], the P-COOH-CS coupled with GTMAC as an antibacterial material [23], the P-COOH-CS-RG19 nanofiber membrane for soluble microbial products removal, protein purification, dyed nanofiber coupled with PHMB for antibacterial material [17]. Therefore, the functionalized nanofiber membrane obtained from the PAN nanofiber modification process has considerable applications. The maximum adsorption capacity for lysozyme using RG19 immobilized onto the CS modified nanofiber membrane was found to be relatively higher than those for lysozyme using the conventional adsorbers [14,15,16,51,52,53,54,55,56,57,58], such as the P-CS-RB2, P-EDA-RB49, P-CS-RB49, and P-CS-RO4 dyed nanofiber membranes. A comparison of the adsorption capacities of various adsorbers for lysozyme in batch modes was shown in Table 6. Overall, the P-COOH-CS-RG19 nanofiber membrane has a high potential for the removal of soluble proteins from biological wastewater in batch and continuous membrane chromatography.

## 4. Conclusions

This study presented the immobilization of waste dyes on the chitosan-modified nanofiber and the dyed nanofiber was an inexpensive functionalized material to treat soluble proteins from biological wastewater. The capture of lysozyme by dyed nanofiber membrane is mainly controlled by electrostatic and ion exchange interactions. The adsorbed lysozyme by the dyed nanofiber membrane can be completely eluted by 1 M of NaCl. The kinetic and equilibrium isotherm studies for adsorption of lysozyme from dyed nanofiber were well described by the pseudo-second-order kinetic model and the Langmuir isotherm model, respectively. The maximum adsorption capacity for lysozyme was increased with increasing the operating temperature. The adsorption capacity for lysozyme was approximately 1293.66 mg/g at a temperature of 318 K, which was much higher than other conventional membranes. The optimum conditions allowed maximum adsorption capacity of lysozyme by nanofiber membrane due to the optimized molecular weight of CS, which significantly affects the adsorption by nanofiber membrane. The P-COOH-CS-RG19 nanofiber appears to be a promising material to remove biomacromolecules from biological wastewater and renders the regeneration of dyed nanofiber for reuse.

## Figures and Tables

**Table 1:** Carboxyl and amine contents of nanofiber membranes.

Membranes	μmol−COOH/g Membrane	μmol−NH_2_/g Membrane
P-COOH	261.66	-
P-COOH-CS (50 kDa)	0.87	179.06
P-COOH-CS (210 kDa)	1.43	145.39
P-COOH-CS (340 kDa)	7.65	112.65

**Table 2:** Pseudo-first-order, pseudo-second-order, and Elovich kinetic rate constants, calculated and experiment *q_exp_* values for the removal of the P-COOH-CS-CEW nanofiber membrane at different dye concentrations.

Lysozyme (mg/mL)	Pseudo-First-Order			Pseudo-Second-Order				Elovich		
	*k_1_* (mg/g·min^1/2^)	*q_1_* (mg/g)	*R^2^*	*k_1_*	*q_cal_* (mg/g)	*q_exp_* (mg/g)	*R^2^*	*α*	*β*	*R^2^*
0.5	0.0273	176.35	0.5794	1.54 × 10^−3^	181.82	185.60	0.9981	907.5200	0.04780	0.7741
1.0	0.0285	356.48	0.9092	4.61 × 10^−4^	370.37	367.69	0.9970	342.2725	0.01855	0.8838
2.0	0.0271	598.66	0.9455	1.80 × 10^−4^	625.12	610.90	0.9963	183.6610	0.00892	0.9243

**Table 3:** Kinetic values calculated by intraparticle diffusion kinetic model for lysozyme adsorption onto P-COOH-CS-RG19 nanofiber membrane at different dye concentrations.

Lysozyme (mg/mL)	Intraparticle Diffusion Model					
	First Stage	Second Stage			Third Stage	
	*k_p_* (mg/g∙min^1/2^)	*R^2^*	*k_p_* (mg/g∙min^1/2^)	*R^2^*	*k_p_* (mg/g∙min^1/2^)	*R^2^*
0.5	32.73	0.9945	8.312	0.7691	1.165	0.2390
1.0	55.59	0.9940	18.52	0.9134	2.581	0.4035
2.0	82.14	0.9943	23.15	0.9506	5.534	0.6602

**Table 4:** Equilibrium and thermodynamic parameters calculated using equilibrium isotherm models for determining the adsorption behavior of P-COOH-CS-RG19 nanofiber membrane for lysozyme.

Temperature	Langmuir			Freundlich			Temkin isotherm		
(K)	*q_max_* (mg/g)	*K_d,_* (mg/mL)	*R^2^*	*n_F_*	*K_d,F_*	*R^2^*	*b*	*K_T_* (mg/mL)	*R^2^*
288	648.5084	0.01309	0.9973	38.1679	669.1444	0.1132	144.9414	4.0832 × 10^17^	0.1174
298	708.7172	0.05489	0.9881	4.8685	660.5018	0.7697	30.1041	2874.2104	0.8537
318	1293.6610	0.07280	0.9964	7.2254	1207.1288	0.8820	17.4281	2853.9671	0.8729

**Table 5:** The thermodynamic parameters for the adsorption of lysozyme onto the P-COOH-CS-RG19 nanofiber membrane at different temperatures.

Temperature	*K_d_*	*K_ads_*	Δ*G^0^_ads_*	Δ*H^0^_ads_*	Δ*S^0^_ads_*
(K)	(mg/mL)	(mL/mg)	(kJ/mol)	(kJ/mol)	(kJ/mol)
288	0.01309	76.3941	−10382.0337	−39641.1520	10244.3909
298	0.05489	18.2182	−7190.9646		7057.9407
318	0.07280	13.7362	−6926.9962		6802.3385

**Table 6:** Comparison with conventional membranes for adsorption capacity of pure lysozyme.

Membranes	Maximum Binding Capacity (mg/g)	Maximum Binding Capacity (mg/cm^2^)	References
P-COOH-CS-RG19	769.23	4.70	This work
PAN-COOH-CS-RB2 nanofiber membrane	245.58	1.50	[14]
P-COOH-CS-RO4 nanofiber membrane	369.92	2.26	[15]
P-COOH-EDA-RB49 nanofiber membrane	587.25	3.59	[16]
P-COOH-CS-RB49 nanofiber membrane	416.67	2.54	[16]
Reactive Blue 4 immobilized onto pHEMA/chitosan membranes.	21.67	1.64	[54]
Reactive Red 120 immobilized pHEMA/chitosan composite membrane	31.75	2.40	[54]
Procion Green H4-G immobilized pHEMA/chitosan composite membrane	63.66	4.81	[53]
Procion Brown MX-5BR immobilized pHEMA/chitosan composite membrane	48.06	3.63	[53]
Procion Brown MX-5BR immobilized pHEMA/chitosan IPNs membrane	101.75	3.33	[52]
Procion Green H-E4BD immobilized pHEMA ion exchange membrane	11.22	0.42	[51]
Reactive Green 19 immobilized pHEMA/chitosan composite membrane	48.25	1.97	[55]
Procion Brown MX-5BR immobilized pHEMA/chitosan composite membrane	96.43	3.16	[56]
Procion Green H-4G immobilized pHEMA membrane	63.65	2.19	[57]
P-COOH-Tris nanofiber membrane	345.83	2.11	[58]

## Data Availability

Due to the nature of this research, participants in this study did not agree for their data to be shared publicly, so supporting data are not available.
